# Prevalence, Determinants, and Consumer Stance towards Dietary Supplements According to Sex in a Large Greek Sample: A Cross-Sectional Study

**DOI:** 10.3390/nu14235131

**Published:** 2022-12-02

**Authors:** Panagiotis-David Soukiasian, Zacharenia Kyrana, Konstantina Gerothanasi, Efstratios Kiranas, Lambros E. Kokokiris

**Affiliations:** 1 Department of Nutritional Sciences and Dietetics, School of Health Sciences, International Hellenic University, 57400 Thessaloniki, Greece; 2 Laboratory of Agronomy, Faculty of Agriculture, Forestry and Natural Environment, School of Agriculture, Aristotle University of Thessaloniki, 54124 Thessaloniki, Greece

**Keywords:** dietary supplements, prevalence, determinants, consumer stance, Greece

## Abstract

A stratified random sampling technique was used in order to explore the prevalence of and the factors influencing dietary supplement (DS) use and the consumer stance towards DS by Greeks (*n* = 28,491, ≥15 years) according to sex. Additionally, we performed a Pearson’s chi-square to test within DS users for the dependence between sex and the examined factors, and binary logistic regression to create predictive DS user profiles. A history of DS use accounted for 55.5% and was more prevalent in women (58.4% vs. 52.3%, *p* < 0.001); multivitamins were the most popular DS used. The significant predictors for DS use for both sexes were age, education, employment status, exercise, and following a special diet, while BMI and monthly income were significant for women and men, respectively. The women and men DS users had mostly illness-health and fitness-related goals, respectively. Substantial proportions of the respondents had false beliefs about DS. A noticeable percentage of DS users displayed imprudent behavior regarding DS use, e.g., one out of five did not know the definition or did not take into consideration the recommended daily allowance. While several factors seemed to impact DS use, with certain differences between the sexes, the considerable lack of knowledgeable and responsible use of DS, with few differences between the sexes, observed can point state authorities and health professionals towards appropriate countermeasures.

## 1. Introduction

“Let food be thy medicine and medicine be thy food” is a popular quote that is frequently attributed to Hippocrates (400 BC). However, in which category do dietary supplements (DS) fit? In legal terms, DS are defined as “foodstuffs”, but they can come in many forms that are similar to those of medicines, such as tablets or pills; yet, manufacturers of DS, unlike those of drugs, are not allowed to claim the treatment or prevention of diseases, since the purpose of DS is to “supplement the normal diet” and to produce a “nutritional or physiologic effect” [1]. However, it is worth noting that DS are not as meticulously regulated as drugs and they are much more accessible to consumers (e.g., via supermarkets) [2].

Due to these factors, it is questionable whether the consumer is in the position to pragmatically tell the difference and make appropriate decisions, while the imprudent use of DS can lead to adverse outcomes (e.g., DS–drug interaction [3] or toxicity), just as much a drug can. Nonetheless, in this context, it would be an oversight to not mention that DS, indeed, can prove to be essential and can even be recommended for certain cases, such as groups of people who are at risk for nutritional deficiencies (e.g., vitamin B12 for animal-excluding diets [4] or folate for pregnant/lactating women [5]).

With that in mind, the current literature on nationwide data indicates a pronounced popularity of DS use among the general population on a global scale; yet, certain differences are noted depending on the country, the usage timeframe that is explored (e.g., “have you used a DS during the last *x* hours/days/weeks etc.?”), and the point in time when such information was collected. For instance, before the COVID-19 pandemic (i.e., March 2020 [6]), the percentage of those who used a DS during the last 12 months prior to their questioning was ~10% in both Spain [7] and Poland [8], but ~65% in Lithuania [9]. However, after COVID-19 was established as a pandemic, those percentages increased to 21% [10], ≥48% (“at least once in lifetime usage”) [11], and ~80% [9] respectively, indicating that COVID-19 might have played an impactful role.

Such a shift among Greek consumers was recently shown to be insignificant, with roughly one in three having used a DS in the previous two weeks [12]; yet, a recent Greek study showed that ~35% and ~20% of its participants had shifted towards healthier nutrition and started/increased their DS consumption in order to enhance their immune system, respectively [13]. However, besides these two recent Greek studies, to our knowledge only two other studies have examined the usage of DS in Greece; namely, the European Prospective Investigation into Cancer and Nutrition (EPIC) study, which found a usage rate of 2% for men and 6.7% in women (24 h prior) between 1995 and 2000 [14]. In addition, a study by Lidell et al. in 2007 on a convenience sample of Greek women who were around 26 years old found an “at least once per week” usage rate of around 27% [15].

Meanwhile, beliefs that DS are generally harmless seem to be established among many consumers [16], and DS users seem to have a variety of expectations for DS use, ranging from an improvement [17] or taking care of their overall health [18] to enhancing their appearance [19]. However, motivations that were aimed towards the immune system rose during the pandemic [9,11]. On the other hand, others might distance themselves from DS use for reasons such as a lack of respective deficiencies or because of concerns for their safety [20].

Having considered the above, it is underlined that several demographic factors have been identified that influence the consumption of DS, such as sex [12,21], education [7,22], and level of physical activity [20,23].

Therefore, by taking into consideration the scarcity of relevant studies regarding DS use in Greece, especially in the pre-COVID-19 pandemic period, the research questions of this pre-COVID-19 study are the following: (i) What are the occurrence rates of the personal and demographic characteristics, the DS use, the awareness of what DS are, the views and the knowledge about DS use, the reasons for DS use and non-use, and the types of DS that are used by Greek consumers, overall and according to sex? (ii) Is there an independence between or within the sexes and the outcomes from the aforementioned topics when examining DS users, non-users, or both overall? (iii) What are the factors contributing to the profile of a DS user in terms of increasing the possibilities of DS use in Greek men and women? The above will provide a map that will be able to guide state authorities and health care professionals for the improved implementation of relevant decisions, and act as a reference point for a comparison with future homologous studies either during or after COVID-19.

## 2. Material and Methods

### 2.1. Study Design

The present study was based on questionnaires, which were collected during the period from 2018 to 2019, as part of the Development Project “Creation of a database in the Department of Nutrition and Dietetics, to investigate the Nutrition Habits of Greek consumers and their relationship with the Nutrition Supplements and the Nutrition Label”, at the Department of Nutrition and Dietetics of the International Hellenic University.

The in-person questionnaires were administered by the members of the NUTSTUDY team (90 trained senior students of the Department of Nutritional Sciences and Dietetics-International Hellenic University (IHU) and their professors). The approval of the research protocol was provided by the Committee for Research Ethics (IHU). The consent of the participants of this study was given verbally to the senior students, after providing the participants with a research information sheet and informing them about the purpose of the study; thereafter, they were given instructions on how to fill out the questionnaire correctly. The senior students were trained through special lectures.

The target population was the Greek population, and the sample collection method was proportional stratified random sampling. Specifically, the Greek population was divided into the 74 regional units (strata) corresponding to the 13 administrative regions of Greece. The senior students visited food stores, supermarkets, gyms, pharmacies etc., at each regional unit, distributing the questionnaire randomly to people aged 15 and older, without taking into consideration socio-economic, educational, and other inclusion criteria.

The collected sample was representative of the general Greek population in terms of sex. For this reason, the statistical comparisons and the creation of the DS user profiles were based on sex. Specifically, according to the 2011 census, the Greek population consisted of 51% women and 49% men. Accordingly, our sample consisted of 53% women and 47% men.

The initial sample size consisted of 31,824 Greek citizens. The questionnaires that were incomplete were removed. Hence, the final sample size consisted of 28,491 respondents.

The aim of this study was to investigate the attitudes of citizens towards DS use in Greece. Specifically, the frequency and reasons for use/non-use of DS, types of DS, and the respondents’ knowledge, opinions, and behaviors towards DS were examined. The questionnaire consisted of 32 closed-ended questions, which were divided into 3 basic sections. The first section was about the personal and demographic characteristics of the participants, the second section was about the knowledge about DS, and the third section included questions related only to DS users. The assumed definition of DS was as follows: “foodstuffs the purpose of which is to supplement the normal diet and which are concentrated sources of nutrients or other substances with a nutritional or physiological effect, alone or in combination, marketed in dose form, namely forms such as capsules, pastilles, tablets, pills and other similar forms, sachets of powder, ampoules of liquids, drop dispensing bottles, and other similar forms of liquids and powders designed to be taken in measured small unit quantities” (Directive 2002/46/EC, European Parliament and Council, 2002) [1].

### 2.2. Statistical Analysis

For all of the variables, the frequencies and percentages were presented overall and according to sex, age, body mass index (BMI), monthly income, education level, employment status, physical activity, and DS use. Pearson’s chi-square test was used to detect the existence (or nonexistence) of statistically significant dependence between subgroups of the categorical variables [24]. In addition, binary logistic regression analysis (BLR) was performed [25], while all of the analysis’ conditions were examined, in order to create a general predictive profile of a DS user in Greece. The dependent variable was the binary variable of DS use, having two mutually exclusive and exhaustive categories (“Non-user” and “User”). The reference category of the dependent variable was the DS users. The independent variables were the nominal variables of the personal, demographic, and social characteristics of Greek respondents (i.e., sex, age, BMI, monthly income, education level, profession, exercise, and type of diet). All of the assumptions of the BLR were examined and were valid. The statistical analysis was performed using IBM SPSS Statistics v26.0. The significance level was set at α = 0.05.

## 3. Results

### 3.1. Demographic Characteristics

A significant dependence between the personal and the demographic characteristics and the sex of the respondents was observed (*p* < 0.05, Table 1). The majority of the respondents were women (*n* = 15,109, 53%). The most common age of the respondents was 21–30 years old (37% overall, 38% vs. 36% for men and women respectively). In addition, the overall majority (54.4%) had normal weight (45.3% vs. 62.4%), while 32.6% were overweight (42.7% vs. 23.6%). Most of the respondents had up to 1000 euros monthly income (80.9%), while 19.1% had more than 1000 euros. They were school or university students (25.7%), private or public employees (25.2% and 13.7%, respectively), freelancers or farmers (16.8% and 4.7%, respectively), and unemployed (13.9%). The majority (56.5%) were exercisers (61.6% vs. 51.9%). The main diets were the mixed unrestricted diet (67% overall, 70.2% vs. 64.2% according to sex) and the fat-restricted diet (15.3%, 15.4% vs. 15.1%).

### 3.2. DS Use According to Sex

The use of DS was more prevalent among women compared to men (58.38% vs. 52.23%). A low percentage (1.4%) declared that they could not remember if they had used a DS. The DS use was influenced by all of the demographic factors that were examined for both sexes, except for BMI in women (*p* = 0.077, Table 2). Specifically, in the men, the usage was more prevalent in the age groups of 21–30 (57.7%) and 31–40 (57.3%), in all of the BMI categories, except for obesity (47.9%), among those with a monthly income of >500 euros (55.1%–60.1%), in all of the education levels, except for primary education (29.5%), in all of the categories of employment status, except for “unemployed” (43.5%) and “farmers” (39.4%), in the exercisers (59.4%), and among those who followed any type of diet, except for mixed unrestricted (48.2%). With regards to the women, the DS use was more pronounced in all of the age groups, except those between 15 and 20 years old (47.2%) and those >60 years old (49.8%), among those with a monthly income of >500 euros (61.8%–64.8%), in all of the education levels, except for primary education (41.1%), in all of the categories of employment status, except for “unemployed” (48.3%) and “farmers” (48.3%), in the exercisers (61.4%), and among those who followed any type of diet (Table 2).

### 3.3. The Awareness of What DS Are and Views about Their Use

The awareness of what DS are and the views about DS use are presented in Table 3. A significantly higher proportion of women, compared to men, knew what DS are (90.4% vs. 87.0%, *p* < 0.001), with this difference existing for both women DS users (94.6% vs. 93.5%, *p* < 0.01) and non-users (84.3% vs. 79.9%, *p* < 0.001). Additionally, women overall, compared to men (24.0% vs. 26.0%, *p* < 0.001), and women DS users compared to men DS users (27.1% vs. 32.4%, *p* < 0.001), believed less frequently that DS are generally harmless. In addition, women DS users believed significantly less frequently than men DS users that regular DS use can prevent many ailments (25.0% vs. 26.5%, *p* < 0.05).

### 3.4. The Reasons for DS Non-Use

The reasons for DS non-use are presented only for DS non-users in Table 4. The top reasons for non-usage were the fear of side effects (37.2%), a good level of fitness (34.1%), and the adherence to a proper diet (33.2%). More specifically, there was a significant dependence between some of the reasons and the sex of the respondents, namely the satisfactory level of fitness (36.1% vs. 32.1%, *p* < 0.001) and other reasons (7.3% vs. 8.7%, *p* = 0.004), such as an absence of necessity (2.9% vs. 3.8%, *p* = 0.008), indifference or a lack of consideration (0.6% vs. 1.0%, *p* = 0.012), and “not being informed by a doctor” (0.4% vs. 0.7%, *p* = 0.012).

### 3.5. The Reasons for Use

The remaining part of the statistical analysis concerns only the DS users. The reasons for DS use (for only the DS users) are presented in Table 5. There was a statistically significant dependence between sex and the reasons for DS use, such as the improvement of physical condition (47.7% vs. 33.3%, *p* < 0.001) and the treatment of nutrient deficiencies (30.4% vs. 40.2%, *p* < 0.001) and pathological conditions (16.7% vs. 31.3%, *p* < 0.001). However, significant dependence is not noted regarding the prevention of health problems (21.5% vs. 22.5%, *p* = 0.121). Furthermore, regarding the treatment of pathological conditions, it seems that men had used DS to treat individual pathological conditions, either less frequently (e.g., anemias; 3.3% vs. 15.8%, *p* < 0.001) or at a similar frequency as women (e.g., obesity; 2.5% vs. 2.7%, *p* = 0.373), with the only exception being cardiovascular diseases (CVD) (2.4% vs. 0.9%, *p* < 0.001).

Additionally, the majority (78.8%) of the total DS users declared taking into account the recommended dietary allowance (RDA) of the active ingredients during DS use. However, although the rate of the RDA’s consideration among men and women was similar (78.8% vs. 78.7%, respectively), men more frequently directly declared not taking said RDA into consideration (13.6% vs. 11.3%, *p* < 0.001), even if they knew what RDA meant. On the other hand, women were more likely to not know what RDA meant (10.0% vs. 7.6%, *p* < 0.001). Furthermore, only 48.8% of DS users knew about the risks of their excessive use, with the higher awareness being attributed to men (51.2% vs. 46.8%, *p* < 0.001).

The DS types that have been used are presented in Table 6. The most common types were vitamins (77.3% overall, men: 74.8% vs. women: 79.3%, *p* < 0.001), minerals (54.4% overall, men: 41.3% vs. women: 64.7%, *p* < 0.001), herbs or extracts (50.3% overall, men: 47.3% vs. women: 52.7%, *p* < 0.001), and other unclassified DS types (49.3% overall, men: 63.3% vs. women: 38.1%, *p* < 0.001). Characteristically, women DS users, compared to men, had used DS that contained *Fe* (42.1% vs. 14.2%, *p* < 0.001), vitamin B12 (9.1% vs. 8.1%, *p* = 0.028), and folic acid (16.9% vs. 4.0%, *p* < 0.001) at a higher rate, while they had used *Cu* at a lower rate (0.7% vs. 1.1%, *p* = 0.032). Similarly, women seemed to have used *Ca* (23.0% vs. 10.2%, *p* < 0.001) and vitamin *D* (11.6% vs. 9.3%, *p* < 0.001) more frequently. Lastly, men had used protein (37.6% vs. 6.9%, *p* < 0.001), amino acid (12.4% vs. 3.6%, *p* < 0.001), creatine (17.4% vs. 1.7%, *p* < 0.001), carnitine (9.0% vs. 3.0%, *p* < 0.001), and energy drink DS (12.4% vs. 4.5%, *p* < 0.001) at a higher rate. Nonetheless, based on a chi-square test, it seems that there was no statistically significant dependence between the sex of DS users and the history of usage of DS consisting of biotin (1.5% vs. 1.5%, *p* = 0.748) and a complex of *B* vitamins (9.6% vs. 9.8%, *p* = 0.634), *Se* (1.9% vs. 2.1%, *p* = 0.570), hippophaes (14.0% vs. 14.2%, *p* = 0.801), gingko (2.5% vs. 2.2%, *p* = 0.165), grape extract (1.6% vs. 1.5%, *p* = 0.652), DS for weight loss or fat-burner (8.7% vs. 7.9%, *p* = 0.059), and other DS (1.0% vs. 1.2%, *p =* 0.179).

### 3.6. Binary Logistic Regression Analysis

The results of the final binary logistic regression analysis, with the *Enter* method, for the 13,199 men (who were stated as DS users or non-users) showed that the overall multivariate model was statistically significant, (chi-square test of Pearson, *χ*^2^ = 924.538 and *p* < 0.001) and correctly predicted 61.6% of the cases. Similarly, for the 14,903 women, the results showed that the overall multivariate model was statistically significant, (chi-square test of Pearson, *χ*^2^ = 518.766, *p* < 0.001) and correctly predicted 60.7% of the cases.

The results of the multivariable-adjusted odds ratios (OR) and the 95% confidence intervals (CI) for the association between DS use and the personal, demographic, and social characteristics of both the men and the women are shown in Table 7.

In the men, the type of diet that was adopted, the employment status, the exercise status, the age group, the monthly income, and the education level were the most influential predictors for a history of DS use (*p* < 0.05, Table 7).

Specifically, regarding the type of diet that was adhered to, in comparison with the reference variable (i.e., the men following a mixed unrestricted diet), higher odds (thus a higher possibility of being a DS user) were noted, in a descending order, for those following a vegan/vegetarian diet (111.1%), a starch/carbohydrate-restricted diet (98.0%), a lacto-vegetarian diet (95.6%), a lacto-ovo-vegetarianism diet (65.6%), a fat-restricted diet (62.6%), and a calorie-restricted diet (26.8%). Additionally, in comparison with the unemployed men, higher odds for DS usage were observed, in a descending order, for the private employees (41.4%), the freelancers (36.6%), and the students (17.9%). In addition, the non-exercisers, in comparison with the exercisers, had 46.1% lower odds of using DS. Lastly, in comparison with those aged >60 years old, the odds for DS use were higher in the men aged between 21 and 30 years old (64.8%), between 31 and 40 years old (54.8%), and between 41 and 50 years old (24.8%), while the odds lost their significance for those between 15 and 20 years old and 51 and 60 years old (*p* > 0.05, Table 7).

However, it was also noted that the men with a monthly income of less than 500 euros, in comparison with those attaining more than 2000 euros, had 31.0% lower odds for using DS, with a diminished significance for the income groups in between these groups. Similarly, the men with a primary education, compared with those on a postgraduate level, had 47.8% lower odds for DS usage, while the education levels in between these groups did not attain any statistical significance (*p* > 0.05, Table 7).

In the women, the type of diet that was adopted, the employment status, the exercise status, the education level, the BMI class, and the age group were the most influential predictors for a history of DS use (*p* < 0.05, Table 7). Characteristically, regarding the type of diet that was adhered to, in comparison with the women following a mixed unrestricted diet, higher odds were noted, in a descending order, for those following a vegan/vegetarian diet (53.9%), a starch/carbohydrate-restricted diet (35.4%), a lacto-ovo-vegetarian diet (33.7%), and a fat-restricted diet (18.8%). In addition, in comparison with the unemployed women, higher odds for DS usage were observed, in a descending order, for the freelancers (57.6%), the private (48.9%) and public employees (45.3%), and the students (43.8%). In addition, the non-exercisers, in comparison with the exercisers, had 20.1% lower odds of using DS. In comparison with those who had a postgraduate education level, those below that level of education had lower odds of using DS, but the magnitude of these lower odds was reduced as the level of education increased. Namely, lower odds of DS use were observed for the primary (55.1%), the secondary (24.9%), and the tertiary (12.0%) education levels. In addition, in comparison with those with a normal BMI, higher odds for DS use were noted for the underweight (31.1%) and the obese (28.2%) individuals, while the overweight women did not attain any statistical significance (*p* > 0.05, Table 7). Lastly, in comparison with those aged >60 years old, the odds for DS use were lower in the women aged between 15 and 20 years old (39.2%), while the odds lost their significance for those between 21 and 60 years old (*p* > 0.05, Table 7).

## 4. Discussion

### 4.1. Prevalence of Dietary Supplement Use

Our study found that nine out of ten of the respondents were aware of what DS are, with around 55% (men: 51.6% vs. women: 57.6%) of our sample reporting DS use at least once during their lifetime. However, even though the usage rates vary by country and the period of data collection (in relation to the COVID-19 pandemic), a comparison with different studies is difficult because the usage rates vary by the defined usage timeframe. Thus, before the pandemic, similar studies revealed high percentages of history of DS use (e.g., Switzerland 53% [26] and Saudi Arabia 63.2% [16]); meanwhile, others examined DS usage using a relatively wide usage timeframe, such as during the last one or two years (e.g., Spain: 9.3% [7], Poland: 10% [8], France: 40.8% [27], Italy: 49% [22], Netherlands: 62% [18] and Lithuania: 66.1% [9]) or a narrower range, such as during the previous week/month (e.g., France: 24.8% [27], Greece: 31.4% [12], Australia: 43.2% [28], Denmark: 55.8% [21] and USA: 57.6% [29]). Indeed, many studies have shown an increase in DS use after the pandemic began (e.g., Spain: 21.3% [10], Poland: 48–79% [11] and Lithuania: 78.1% [9]), with Greece, however, showing mixed results [12,13].

### 4.2. Attitudes towards Dietary Supplements

Interestingly, we found that one out of four of the respondents believed that DS are generally harmless. In addition, around 20% of our respondents believed that DS can prevent many ailments, and 15% believed that DS are necessary for all ages. Other studies have also generated significant percentages supporting similar views in individuals both with and without a health science background [16,30,31,32,33,34]. In fact, studies have shown that during the pandemic a certain disease preventative/treatment mindset was shaped amongst many consumers, as they had made a connection between the enhancement of their immune system and DS consumption [9,11,13,20,35,36,37,38,39]; meanwhile, the current literature does not currently advise for or against the use of DS against the prevention or the treatment of COVID-19 [40].

Either way, we found that DS users, compared to non-users, were more likely to have beliefs that were favorable towards the use of DS; a stance that has previously been seen in the literature [41].

Regardless, despite certain statistical differences between the men and women, a significant lack of responsible and knowledgeable use of DS has been observed. Specifically, one out of two DS users did not know the dangers of overuse and around one out of five DS users either did not know what RDA means or directly did not take it into consideration. At the same time, large proportions of DS users, ranging roughly from 20% to 30%, attributed therapeutic and illness-preventative effects to DS, and supported their need for all ages, while around 5% believed that DS can prevent cancer. This sort of behavior can be alarming, as adverse effects of DS can take place even during “regular” or “casual” use (e.g., chemical–chemical, pharmacodynamic, and pharmacokinetic interactions with medications [3] and toxicity in itself).

### 4.3. Reasons for Not Using Dietary Supplements

As a part of a somewhat unexplored field, we examined the reasons for not using DS. Specifically, the top reason for not using DS was the fear of side effects, which was reported by around four out of ten DS non-users, followed by a satisfactory level of fitness and a proper diet, which was expressed by around three out of ten, with “lack of necessity” being cited by merely 3%. Other studies regarding the reasons for DS non-use have reported the top reasons to be an absence of necessity [20,42], a lack of vitamin deficiency [20], and adequate nutrition [43], while concerns about their safety were expressed by comparatively low percentages; namely ~5% for a Middle Eastern general population sample [20] and ~8% for a Polish university student sample [43]. However, it seems that results are not homogeneous between different populations. For example, in a study by Axon et al. on a sample of US pharmacology students, only 0.6% of DS non-users avoided using DS because of their perceived unsafety [42]. Nonetheless, regarding our aforementioned primary reasons for non-use, we found that the only difference between the sexes was for “a satisfactory level of fitness”, as men expressed it more frequently.

### 4.4. Reasons for Using Dietary Supplements

Regarding the reasons for DS use, we found that, overall, the most common motivator was the improvement of physical condition, followed by the treatment of nutrient deficiencies and the treatment and prevention of pathological conditions. Our results are in agreement with similar pre-pandemic studies, which have found the main reasons for use to be “health” [18,44,45], the improvement of “health” [17,43] or “well-being” [46], the prevention or treatment of nutrient deficiencies [19,34], or a medical necessity [34]. However, other reasons, such as stress [47] and fatigue [27], have also been reported to be the prime motivator in certain studies. Nonetheless, one should keep in mind that the “improvement of the immune system” or the “prevention/treatment of COVID-19” as expected outcomes via DS use can be seen more frequently in the literature that was generated during the pandemic [9,11,13,35,36,37,38,39]. Meanwhile, our results indicate that the “motivational hierarchy” between men and women differs. Namely, men demonstrated fitness-related goals more frequently, while women were more illness-health oriented. Specifically, the top reasons for use by men were the improvement of their physical condition, an increase in muscle mass, the restoration of nutrient deficiencies, and an increase in sports performance. On the other hand, the top reasons by women were the restoration of nutrient deficiencies, the improvement of physical condition, and the treatment and prevention of pathological conditions.

Our results are in agreement with similar studies, where one can see that women are comparatively more, or mostly, interested in using DS that are directed towards their wellbeing [46] or health [18,34,44,46] and dealing with [27,34,46,47,48] or the prevention [19] of health issues. However, additional motives, such as “beauty” [44] or “good appearance” [19] and the “maintenance of healthy hair” [48], have been reported. On the other hand, men have been shown to be comparatively more frequently gravitated towards the enhancement of sports performance/exercise [18,27,46,47] and increased muscle mass or energy [34,44]. Indeed, Rontogianni et al., recently showed that it is significantly more probable for a woman to be a DS user if she has a history of chronic disease; however, such an association for men was merely suggestive [12]. In addition, even in a gym-setting, where one could expect little differences between the sexes, it has been noted that women DS users were more likely to exhibit “health-oriented purposes” (i.e., the prevention of deficiencies and the treatment/prevention of disease), while men leaned towards muscular improvement [49]. One should note that, in our study, the women selected either significantly more often or at the same frequency all of the listed pathological conditions, except for “cardiovascular diseases”. The examples that stood out are “anemias”, which was selected five times more often (15.5% vs. 3.3%), and “osteoporosis”, which was selected four times more often (4.4% vs. 1.1%). Interestingly, the specific sex differences regarding these three examples have been reported in the past [17]. Anemias are indeed more prevalent in women, due to their physiology (i.e., menstruation and pregnancy) and/or due to insufficient dietary intake to cover the increased needs [50]. Similarly, being a woman is also considered to be a risk factor for osteoporosis-related fractures [51]. On the other hand, men are more prone to CVD at earlier ages [52].

### 4.5. Dietary Supplements Used

With regards to the reported types of DS that are used, we found that vitamins were the most popular. Specifically, the most frequently used DS were multivitamins (MV) and vitamin *C*. Our results are in agreement with several other studies that were conducted before the pandemic with regards to the popularity of MV [16,18,29,30,31,33,45] and vitamin *C* [18,33,35,44,45], with few exceptions [19,27,43]; even after the onset of the pandemic, the popularity of MV [10] and vitamin *C* [20,35,36] remained high. The consumption of MV might be an attempt to cover the needs for a large extent of nutrients and, therefore, provide a broader sense of safety to the consumers who seek to take care of their general health; oddly enough, the men tended to consume MV more often even though most of them had fitness-related goals This finding is ambivalent, as it disagrees with the current literature [17,18,23,34,47,53], even though these studies seem to pool MV and multiminerals together; either way, even if we followed the same tactic, our results would still disagree. On the other hand, vitamin *C*, is known to contribute to the normal function of the immune system, as well as for its antioxidant activity [54]. Thus, due to the mostly health-oriented goals of women, its use was more frequent carried out by them; yet, the literature has produced mixed results [18,27,44,47]. Additionally, several other key differences were noted between the DS that were consumed by men and women. For instance, women reported having used DS containing iron, folic acid, and vitamin B12 significantly more often. Regarding iron, our results concur with the literature [18,23,27], but mixed results have been observed regarding folic acid and vitamin B12 [17,27,44]. Given the fact that all of these nutrients revolve around anemia [50], this phenomenon may be associated with the much larger proportion of women using DS to treat anemias, as stated earlier. Similarly, women were more likely to use DS containing calcium and vitamin *D*. These differences regarding calcium can be seen in the literature [17,18,23], but less so for vitamin *D*, as mixed results have been generated [17,18,27,44]. This preference by women could easily be traced back to their motivation to treat osteoporosis. On the other hand, men were more likely to use protein, creatine, energy drink, amino acids, and carnitine DS. The higher usage of exercise/physique-related DS amongst men has also been noted elsewhere [18,22,27,34,44]. Likewise, the motivations for the respective DS selection may be traced to their aforementioned mostly fitness-related goals. Lastly, a comparatively higher usage rate by men was noted for DS containing ω-3 fatty acids, fish oils, and potassium. Generally, regular fish consumption is advised, due the protective effect of the ω-3 fatty acids regarding CVD, while the consumption of a potassium rich diet is known to lower blood pressure, which is a risk factor for CVD [55]. A healthy dietary pattern, rather than DS consumption, is strongly recommended in the context of CVD, while due attention is given to DS use in cases prone to nutrient inadequacy [55]. In this context, one should keep in mind that consumers can come into contact with information regarding the above protective effects of the substances that have been mentioned earlier through relevant health claims that can be found on the labels of the DS [54]; thus, they can take the initiative towards their consumption.

### 4.6. Profile of Supplement Users According to Sex

In alignment with the fairly consistent literature [7,9,12,17,18,19,20,21,22,23,27,28,29,35,53,56], we have found that being a woman is a significant determinant of DS use. Therefore, we have described the “statistical profile” of supplement users separately for men and women. Certain determinants of DS use are shared between men and women and concur with the fairly consistent literature, i.e., having a higher level of education [7,9,22,23,27,28,35,53,56], following a special diet [19,26,27,43] or even tending to follow a healthier diet [21,56], and having a physically active lifestyle [17,19,20,22,23,27,28,56]. Although, Rontogianni et al. did not find a significant association between DS use and education and the level of adherence to a Mediterranean diet, they did report a suggestive positive relationship for physical activity, but only for men [12]. Regarding special diets, anyone who follows an animal-excluding diet, such as vegans, could be expected to consume supplements in order to cover their needs for essential nutrients, such as vitamin B12 [4]. However, other special diets could be arbitrarily assigned as attempts for healthier nutrition. In this context, these consumers, and those with an active lifestyle, might consider DS as an essential part of their endeavor to achieve their goals and, thus, incorporate DS into their lifestyle more frequently. Compared to those unemployed, students and employed individuals, except for farmers and men who are public sector workers, were more likely to use DS. However, regarding the relationship between the monthly income and the DS use, we found it to be significantly negative for men with the lowest income (<500 euros/month). Generally, many studies have produced a positive relationship between DS use and a higher income [9,17,22,53], while its relationship with the employment status is not as homogeneous, as positive [9], negative [23], and mixed relationships [22,27] have been generated; e.g., in a French study, the odds for DS usage for students were of the lowest value, and were even lower than those of the unemployed, while we found students to have higher odds than the unemployed for DS usage [27]. Nonetheless, for income, Rontogianni et al. found no significant associations for either men or women, while for employment a significant positive association was established, but only for men [12]. Considering our outcomes, one could argue that, since education, employment, and income constitute elements of an individual’s socio-economic status [57], the beholders of the better end of these constituents can afford and are able to invest themselves more in additional enhancing paths towards their health, i.e., DS consumption, whereas those with a lower socio-economic status might have different priorities, focusing on the more fundamental necessities. In general, increasing age has been positively associated with DS usage [17,18,19,21,23,27,28,29,53,56], with certain exceptions [7,9,22]. We found that young adult to middle-aged men (21–50 years old), but older women (over 60 years old), were more likely to use DS. Our results are not aligned with those of Rontogianni et al. Specifically, they also found that DS usage was more prevalent among younger men, but any significance was absent in a multivariate logistic regression model; yet, for women, there was a positive relationship, but only up until middle-age, becoming negative thereafter [12]. Nonetheless, other studies that distinguished men and women found a positive relationship between DS usage and age [29,53], except for one, where this relationship was negative [22]. A possible explanation for our results can be generated by observing the motives of the DS users. As mentioned earlier, most men DS users had fitness-related goals. In addition, one could argue that such goals would be expressed mostly by younger audiences, thus explaining the higher likelihood of usage amongst these ages. In the same context, women had mostly illness-health-oriented goals, which in turn could be expected to be expressed more frequently as a person gets older and is more prone to illness.

Regarding BMI, we found that underweight and obese women were more likely to use DS, but BMI did not play a significant role in men. In disagreement with our results, generally DS use has been seen to be mostly negatively correlated with BMI [7,22,23,27,28,56], with some studies, namely those in the US [17,53] and Australia [56], associating DS use with normal BMI, while a study in Germany did not find a significant association at all [19]. Our disagreement persists even when studies analyze the sexes independently. Namely, Rontogianni et al. found, roughly speaking, a negative relationship between DS use and BMI for both sexes [12]; the US 2011–2014 NHANES study showed the highest DS use to be among women of normal BMI and overweight men [53]; and finally, an Italian study located the highest use among underweight men, while BMI was not significant for women [22]. A potential explanation for our results could be that individuals with an abnormal BMI are more likely to face a spectrum of pathological conditions and might have a greater desire to prevent or treat them with DS use; these types of expectations from DS were more pronounced in women, hence making women with abnormal BMI more eager to use DS.

## 5. Conclusions

This is one of the limited Greek studies that explores the prevalence of DS use in Greece. However, it is the first study to explore the consumer stance (i.e., the attitudes, behavior, and opinions) towards DS, essentially giving a cross-sectional map, which can be used to guide state authorities and health professionals with regards to the implementation of relevant policies and the provision of improved services, respectively. In addition, being a pre-COVID-19 study, it can be compared with similar studies that are conducted in the future in order to observe the relevant changes and trends.

A high prevalence of a history of DS use and several factors that influence have been identified in a Greek sample. However, what stands out is a relatively pronounced lack of understanding regarding proper DS use and its associated dangers, as several false beliefs and attitudes have been identified substantially. Such behaviors should be considered dangerous, and thus filling in the knowledge gaps of the public is necessary in order to prevent severe outcomes due to imprudent DS use.

## Figures and Tables

**Table 1:** Absolute and relative frequencies in parenthesis (%) of personal and demographic characteristics overall (total) and according to sex. The *p*-values of chi-square tests are presented.

Category	Total (*n* = 28,491)	Men (*n* = 13,382)	Women (*n* = 15,109)	*p*-Value
**Age** (years old)				<0.001
15–20	3910 (13.7)	1774 (13.3)	2136 (14.1)	
21–30	10,545 (37.0)	5108 (38.2)	5437 (36.0)	
31–40	5892 (20.7)	2861 (21.4)	3031 (20.1)	
41–50	4236 (14.9)	1868 (14.0)	2368 (15.7)	
51–60	2677 (9.4)	1228 (9.2)	1449 (9.6)	
>60	1231 (4.3)	543 (4.1)	688 (4.6)	
**BMI**				<0.001
Underweight	848 (3.0)	108 (0.8)	740 (4.9)	
Normal weight	15,492 (54.4)	6058 (45.3)	9434 (62.4)	
Overweight	9274 (32.6)	5708 (42.7)	3566 (23.6)	
Obese	2877 (10.1)	1508 (11.3)	1369 (9.1)	
**Monthly income** (euros)				<0.001
<500	12,543 (44.0)	5014 (37.5)	7529 (49.8)	
501–1000	10,517 (36.9)	5047 (37.7)	5470 (36.2)	
1001–1500	4057 (14.2)	2392 (17.9)	1665 (11.0)	
1501–2000	812 (2.9)	515 (3.8)	297 (2.0)	
>2000	562 (2.0)	414 (3.1)	148 (1.0)	
**Education level**				<0.001
Primary education	1410 (4.9)	618 (4.6)	792 (5.2)	
Secondary education	11,824 (41.5)	6003 (44.9)	5821 (38.5)	
Tertiary education	12,758 (44.8)	5564 (41.6)	7194 (47.6)	
Postgraduate education	2499 (8.8)	1197 (8.9)	1302 (8.6)	
**Employment status**				<0.001
Unemployed	3972 (13.9)	1396 (10.4)	2576 (17.0)	
Student	7316 (25.7)	3214 (24.0)	4102 (27.1)	
Private employee	7174 (25.2)	3462 (25.9)	3712 (24.6)	
Public employee	3897 (13.7)	1730 (12.9)	2167 (14.3)	
Freelancer	4779 (16.8)	2771 (20.7)	2008 (13.3)	
Farmer	1353 (4.7)	809 (6.0)	544 (3.6)	
**Exercise**				<0.001
Exercisers	16,093 (56.5)	8244 (61.6)	7849 (51.9)	
Non-exercisers	12,398 (43.5)	5138 (38.4)	7260 (48.1)	
**Type of diet**				<0.001
Mixed unrestricted	19,101 (67.0)	9394 (70.2)	9707 (64.2)	
Fat restricted	4348 (15.3)	2064 (15.4)	2284 (15.1)	
Calorie restricted	2742 (9.6)	988 (7.4)	1754 (11.6)	
Starch/carbohydrate restricted	1099 (3.9)	491 (3.7)	608 (4.0)	
Lacto-ovo-vegetarianism	508 (1.8)	199 (1.5)	309 (2.0)	
Vegan/vegetarian	449 (1.6)	158 (1.2)	291 (1.9)	
Lacto-vegetarianism	203 (0.7)	66 (0.5)	137 (0.9)	
Other diet	41 (0.1)	22 (0.2)	19 (0.1)	

**Table 2:** Absolute and relative frequencies in parenthesis (%) of personal and demographic characteristics according to sex and DS use. The *p*-values of chi-square tests are presented.

Category	Men (*n* = 13,199)			Women (*n* = 14,903)		
	Not DS User (*n* = 6292)	DS User (*n* = 6907)	*p*-Value	Not DS User (*n* = 6202)	DS User (*n* = 8701)	*p*-Value
**Age**			<0.001			<0.001
15–20	970 (55.1)	790 (44.9)		1110 (52.8)	991 (47.2)	
21–30	2142 (42.3)	2925 (57.7)		2230 (41.4)	3160 (58.6)	
31–40	1209 (42.7)	1623 (57.3)		1068 (35.6)	1935 (64.4)	
41–50	935 (50.6)	912 (49.4)		903 (38.5)	1441 (61.5)	
51–60	706 (59.7)	476 (40.3)		565 (39.9)	850 (60.1)	
>60	330 (64.6)	181 (35.4)		326 (50.2)	324 (49.8)	
**BMI**			<0.001			0.077
Underweight	49 (45.8)	58 (54.2)		282 (38.6)	448 (61.4)	
Normal weight	2757 (46.0)	3234 (54.0)		3942 (42.3)	5386 (57.7)	
Overweight	2715 (48.3)	2906 (51.7)		1451 (41.3)	2060 (58.7)	
Obese	771 (52.1)	709 (47.9)		527 (39.5)	807 (60.5)	
**Monthly income** (euros)			<0.001			<0.001
<500	2568 (51.7)	2400 (48.3)		3403 (45.9)	4011 (54.1)	
501–1000	2222 (44.9)	2726 (55.1)		2064 (38.2)	3345 (61.8)	
1001–1500	1106 (46.7)	1262 (53.3)		577 (35.2)	1063 (64.8)	
1501–2000	233 (46.0)	273 (54.0)		104 (35.5)	189 (64.5)	
>2000	163 (39.9)	246 (60.1)		54 (36.7)	93 (63.3)	
**Education level**			<0.001			<0.001
Primary education	415 (70.5)	174 (29.5)		442 (58.9)	309 (41.1)	
Secondary education	2890 (49.0)	3012 (51.0)		2529 (44.2)	3198 (55.8)	
Tertiary education	2519 (45.6)	3005 (54.4)		2803 (39.3)	4332 (60.7)	
Postgraduate education	468 (39.5)	716 (60.5)		428 (33.2)	862 (66.8)	
**Employment status**			<0.001			<0.001
Unemployed	774 (56.5)	597 (43.5)		1296 (51.7)	1213 (48.3)	
Student	1550 (48.5)	1649 (51.5)		1787 (44.1)	2268 (55.9)	
Private employee	1415 (41.4)	2000 (58.6)		1387 (37.7)	2292 (62.3)	
Public employee	861 (50.8)	835 (49.2)		764 (35.7)	1378 (64.3)	
Freelancer	1219 (44.5)	1518 (55.5)		695 (34.9)	1295 (65.1)	
Farmer	473 (60.6)	308 (39.4)		273 (51.7)	255 (48.3)	
**Exercise**			<0.001			<0.001
Exercisers	3307 (40.6)	4845 (59.4)		2998 (38.6)	4775 (61.4)	
Non-exercisers	2985 (59.1)	2062 (40.9)		3204 (44.9)	3926 (55.1)	
**Type of diet**			<0.001			<0.001
Mixed unrestricted	4792 (51.8)	4466 (48.2)		4170 (43.5)	5408 (56.5)	
Fat restricted	765 (37.5)	1273 (62.5)		852 (37.8)	1403 (62.2)	
Calorie restricted	424 (43.4)	554 (56.6)		710 (41.3)	1008 (58.7)	
Starch/carbohydrate restricted	153 (31.4)	334 (68.6)		207 (34.3)	396 (65.7)	
Lacto-ovo-vegetarianism	77 (39.1)	120 (60.9)		110 (35.8)	197 (64.2)	
Vegan/vegetarian	50 (32.3)	105 (67.7)		97 (33.7)	191 (66.3)	
Lacto-vegetarianism	24 (37.5)	40 (62.5)		51 (37.8)	84 (62.2)	
Other diet	7 (31.8)	15 (68.2)		5 (26.3)	14 (73.7)	

**Table 3:** Absolute and relative frequencies in parenthesis (%) of the awareness of what DS are and views about DS use according to sex and DS use. The *p*-values of chi-square tests are presented.

	Overall	Men				Women			
	Total	Total	Non-User	User	*p*-Value	Total	Non-User	User	*p*-Value
**Awareness of what DS are Statements**	24,952 (88.8)	11,486 (87.0) **^†^**	5028 (79.9) **^a^**	6458 (93.5) **^c^**	<0.001	13,466 (90.4)	5231 (84.3)	8235 (94.6)	<0.001
DS are necessary for all ages	4061 (14.5)	1901 (14.4)	522 (8.3)	1379 (20.0)	<0.001	2160 (14.5)	527 (8.5)	1633 (18.8)	<0.001
DS are generally harmless	7012 (25.0)	3429 (26.0) **^†^**	1190 (18.9)	2239 (32.4) **^d^**	<0.001	3583 (24.0)	1222 (19.7)	2361 (27.1)	<0.001
Regular DS use can prevent many ailments	6083 (21.6)	2880 (21.8)	1053 (16.7)	1827 (26.5) **^e^**	<0.001	3203 (21.5)	1025 (16.5)	2178 (25.0)	<0.001
DS can prevent cancer	1276 (4.5)	629 (4.8)	267 (4.2) **^b^**	362 (5.2)	0.007	647 (4.3)	217 (3.5)	430 (4.9)	<0.001
None of the above	13,509 (48.1)	6212 (47.1) **^‡^**	3856 (61.3)	2356 (34.1) **^d^**	<0.001	7297 (49.0)	3762 (60.7)	3535 (40.6)	<0.001

**^†,‡^** Regarding DS users and non-users overall: statistically significant difference between men and women, overall, with **^†^**: *p* < 0.001 and **^‡^**: *p* < 0.01). ^**a**,**b**^ Regarding DS non-users: statistically significant difference between men and women, with **^a^**: *p* < 0.001 and **^b^**: *p* < 0.05); ^**c**,**d**,**e**^ Regarding DS users: statistically significant difference between men and women, with **^c^**: *p* ≤ 0.01, **^d^**: *p* < 0.001, and **^e^**: *p* < 0.05.

**Table 4:** Absolute and relative frequencies in parenthesis (%) of the reasons for DS non-use overall and according to sex. The *p*-values of chi-square tests are presented.

Category	Total (*n* = 12,494)	Men (*n* = 6292)	Women (*n* = 6202)	*p*-Value
**Side effects fear**	4654 (37.2)	2304 (36.6)	2350 (37.9)	0.141
**Fitness**	4263 (34.1)	2272 (36.1)	1991 (32.1)	<0.001
**Proper diet**	4143 (33.2)	2058 (32.7)	2085 (33.6)	0.280
**Other reasons**	995 (8.0)	457 (7.3)	538 (8.7)	0.004
No necessity	418 (3.3)	184 (2.9)	234 (3.8)	0.008
Opposed to receiving DS	170 (1.4)	96 (1.5)	74 (1.2)	0.109
DS are unknown	106 (0.8)	58 (0.9)	48 (0.8)	0.368
Indifference/lack of consideration	105 (0.8)	40 (0.6)	65 (1.0)	0.012
Untrustworthiness	84 (0.7)	37 (0.6)	47 (0.8)	0.246
“Not being informed by a doctor”	66 (0.5)	23 (0.4)	43 (0.7)	0.012
Financial cost	46 (0.4)	19 (0.3)	27 (0.4)	0.218

**Table 5:** Absolute and relative frequencies in parenthesis (%) of the reasons for DS use overall and according to sex. The *p*-values of chi-square tests are presented.

Category	Total (*n* = 15,608)	Men (*n* = 6907)	Women (*n* = 8701)	*p*-Value
**Improving physical condition**	6192 (39.7)	3293 (47.7)	2899 (33.3)	<0.001
**Nutrient deficiency**	5599 (35.9)	2101 (30.4)	3498 (40.2)	<0.001
**Pathological conditions**	3875 (24.8)	1152 (16.7)	2723 (31.3)	<0.001
Anemias	1602 (10.3)	231 (3.3)	1371 (15.8)	<0.001
Allergies and flues	729 (4.7)	304 (4.4)	425 (4.9)	0.155
Osteoporosis	464 (3.0)	77 (1.1)	387 (4.4)	<0.001
Obesity	404 (2.6)	170 (2.5)	234 (2.7)	0.373
Arthritis	397 (2.5)	122 (1.8)	275 (3.2)	<0.001
Thyroiditis	338 (2.2)	62 (0.9)	276 (3.2)	<0.001
Hypertension	301 (1.9)	149 (2.2)	152 (1.7)	0.064
Digestive	249 (1.6)	90 (1.3)	159 (1.8)	0.009
Cardiovascular	243 (1.6)	167 (2.4)	76 (0.9)	<0.001
Diabetes	218 (1.4)	92 (1.3)	126 (1.4)	0.539
Hyperlipoproteinemia	102 (0.7)	49 (0.7)	53 (0.6)	0.440
Autoimmune disorder	92 (0.6)	27 (0.4)	65 (0.7)	0.004
**Prevention of health problems**	3439 (22.0)	1482 (21.5)	1957 (22.5)	0.121
**Increase in muscle mass**	2492 (16.0)	2140 (31.0)	352 (4.0)	<0.001
**Increase in sports performance**	2439 (15.6)	1851 (26.8)	588 (6.8)	<0.001
**Weight loss**	2089 (13.4)	811 (11.7)	1278 (14.7)	<0.001
**Improving mental function**	1950 (12.5)	772 (11.2)	1178 (13.5)	<0.001
**Pregnancy/birth**	1581 (10.1)	58 (0.8)	1523 (17.5)	<0.001
**Aesthetics/antiaging**	1289 (8.3)	314 (4.5)	975 (11.2)	<0.001
**Enhancement of sexual activity**	375 (2.4)	280 (4.1)	95 (1.1)	<0.001
**Other reasons**	247 (1.6)	78 (1.1)	169 (1.9)	<0.001

**Table 6:** Absolute and relative frequencies in parenthesis (%) of DS types used by DS users overall and according to sex. The *p*-values of chi-square tests are presented.

Category	Total (*n* = 15,608)	Men (*n* = 6907)	Women (*n* = 8701)	*p*-Value
**Vitamins**	12,061 (77.3)	5163 (74.8)	6898 (79.3)	<0.001
Multivitamin	7281 (46.6)	3507 (50.8)	3774 (43.4)	<0.001
Vitamin C	4653 (29.8)	1923 (27.8)	2730 (31.4)	<0.001
Folic acid	1745 (11.2)	277 (4.0)	1468 (16.9)	<0.001
Vitamin D	1645 (10.5)	640 (9.3)	1005 (11.6)	<0.001
B complex vitamin	1518 (9.7)	663 (9.6)	855 (9.8)	0.634
Vitamin B12	1352 (8.7)	560 (8.1)	792 (9.1)	0.028
Vitamin E	1127 (7.2)	449 (6.5)	678 (7.8)	0.002
Vitamin A	954 (6.1)	454 (6.6)	500 (5.7)	0.032
Vitamin B6	550 (3.5)	294 (4.3)	256 (2.9)	<0.001
Vitamin K	488 (3.1)	288 (4.2)	200 (2.3)	<0.001
Niacin	237 (1.5)	129 (1.9)	108 (1.2)	0.001
Biotin	236 (1.5)	102 (1.5)	134 (1.5)	0.748
**Minerals**	8487 (54.4)	2856 (41.3)	5631 (64.7)	<0.001
Iron (Fe)	4643 (29.7)	980 (14.2)	3663 (42.1)	<0.001
Calcium (Ca)	2710 (17.4)	706 (10.2)	2004 (23.0)	<0.001
Magnesium (Mg)	2398 (15.4)	1012 (14.7)	1386 (15.9)	0.028
Mineral complex	1789 (11.5)	858 (12.4)	931 (10.7)	0.001
Potassium (K)	802 (5.1)	464 (6.7)	338 (3.9)	<0.001
Zinc (Zn)	588 (3.8)	305 (4.4)	283 (3.3)	<0.001
Selenium (Se)	314 (2.0)	134 (1.9)	180 (2.1)	0.570
Manganese (Mn)	290 (1.9)	146 (2.1)	144 (1.7)	0.035
Sodium (Na)	261 (1.7)	169 (2.4)	92 (1.1)	<0.001
Chromium (Cr)	154 (1.0)	81 (1.2)	73 (0.8)	0.036
Copper (Cu)	139 (0.9)	74 (1.1)	65 (0.7)	0.032
Cobalt (Co)	118 (0.8)	70 (1.0)	48 (0.6)	0.001
**Herbs or extracts**	7856 (50.3)	3269 (47.3)	4587 (52.7)	<0.001
Green/black tea	3327 (21.3)	1254 (18.2)	2073 (23.8)	<0.001
Spirulina	2724 (17.5)	1147 (16.6)	1577 (18.1)	0.013
Hippophaes	2202 (14.1)	969 (14.0)	1233 (14.2)	0.801
Aloe vera	1790 (11.5)	610 (8.8)	1180 (13.6)	<0.001
Herb combination	1753 (11.2)	743 (10.8)	1010 (11.6)	0.095
Berries	1321 (8.5)	575 (8.3)	746 (8.6)	0.579
Echinacea	1073 (6.9)	347 (5.0)	726 (8.3)	<0.001
Ginseng	903 (5.8)	452 (6.5)	451 (5.2)	<0.001
Garlic	777 (5.0)	411 (6.0)	366 (4.2)	<0.001
Gingko	366 (2.3)	175 (2.5)	191 (2.2)	0.165
Grape extract	243 (1.6)	111 (1.6)	132 (1.5)	0.652
Kava	79 (0.5)	51 (0.7)	28 (0.3)	<0.001
**Unclassified DS**	7691 (49.3)	4373 (63.3)	3318 (38.1)	<0.001
Protein	3199 (20.5)	2599 (37.6)	600 (6.9)	<0.001
Royal jelly	2370 (15.2)	1269 (18.4)	1101 (12.7)	<0.001
Ω-fatty acid	1775 (11.4)	938 (13.6)	837 (9.6)	<0.001
Creatine	1349 (8.6)	1203 (17.4)	146 (1.7)	<0.001
Weight loss/fat-burner	1292 (8.3)	604 (8.7)	688 (7.9)	0.059
Energy drinks	1246 (8.0)	858 (12.4)	388 (4.5)	<0.001
Amino acid	1163 (7.5)	854 (12.4)	309 (3.6)	<0.001
Fish oil	1001 (6.4)	507 (7.3)	494 (5.7)	<0.001
Carnitine	887 (5.7)	622 (9.0)	265 (3.0)	<0.001
Coenzyme Q10	714 (4.6)	357 (5.2)	357 (4.1)	0.002
Glucosamine	286 (1.8)	163 (2.4)	123 (1.4)	<0.001
Other DS	171 (1.1)	67 (1.0)	104 (1.2)	0.179
Melatonin	151 (1.0)	89 (1.3)	62 (0.7)	<0.001
α-Lipoic acid	151 (1.0)	99 (1.4)	52 (0.6)	<0.001

**Table 7:** Wald’s test *p*-values adjusted OR and 95% confidence interval (CI) for the association between men and women DS users the independent variables.

	Men			Women		
Variable	OR (95% CI)	*p*-Value	Overall *p*-Value	OR (95% CI)	*p*-Value	Overall *p*-Value
**Age (years)**			<0.001			<0.001
15–20	1.072 (0.844–1.361)	0.569		0.608 (0.490–0.754)	<0.001	
21–30	1.648 (1.328–2.044)	<0.001		0.895 (0.735–1.089)	0.268	
31–40	1.548 (1.250–1.917)	<0.001		1.191 (0.982–1.444)	0.076	
41–50	1.248 (1.004–1.551)	0.046		1.105 (0.912–1.340)	0.309	
51–60	0.975 (0.776–1.224)	0.827		1.129 (0.926–1.378)	0.230	
>60	Reference			Reference		
**BMI**			0.454			<0.001
Underweight	1.338 (0.902–1.986)	0.148		1.311 (1.119–1.536)	0.001	
Overweight	0.977 (0.903–1.058)	0.566		1.085 (0.996–1.182)	0.062	
Obese	1.002 (0.885–1.136)	0.970		1.282 (1.128–1.458)	<0.001	
Normal weight	Reference			Reference		
**Monthly income (euros)**			0.003			0.995
<500	0.690 (0.548–0.869)	0.002		1.010 (0.712–1.434)	0.954	
501–1000	0.825 (0.664–1.025)	0.083		1.025 (0.725–1.448)	0.890	
1001–1500	0.844 (0.675–1.055)	0.137		1.041 (0.729–1.487)	0.826	
1501–2000	0.880 (0.669–1.157)	0.359		1.013 (0.667–1.539)	0.952	
>2000	Reference			Reference		
**Education level**			<0.001			<0.001
Primary education	0.522 (0.412–0.662)	<0.001		0.449 (0.361–0.559)	<0.001	
Secondary education	0.883 (0.770–1.013)	0.075		0.751 (0.657–0.860)	<0.001	
Tertiary education	0.889 (0.776–1.019)	0.092		0.880 (0.773–1.002)	0.054	
Postgraduate education	Reference			Reference		
**Employment status**			<0.001			<0.001
Student	1.179 (1.024–1.357)	0.022		1.438 (1.276–1.620)	<0.001	
Private employee	1.414 (1.216–1.645)	<0.001		1.489 (1.324–1.676)	<0.001	
Public employee	1.068 (0.897–1.272)	0.458		1.453 (1.258–1.677)	<0.001	
Freelancer	1.366 (1.168–1.598)	<0.001		1.576 (1.375–1.805)	<0.001	
Farmer	1.015 (0.831–1.238)	0.886		1.090 (0.892–1.331)	0.339	
Unemployed	Reference			Reference		
**Exercise**						
Non-exercisers	0.539 (0.499–0.583)	<0.001		0.799 (0.745–0.857)	<0.001	
Exercisers	Reference			Reference		
**Type of diet**			<0.001			<0.001
Fat restricted	1.626 (1.469–1.800)	<0.001		1.188 (1.079–1.309)	<0.001	
Starch/carbohydrate restricted	1.980 (1.620–2.420)	<0.001		1.354 (1.136–1.615)	0.001	
Calorie restricted	1.268 (1.106–1.454)	0.001		1.028 (0.924–1.143)	0.611	
Vegan/vegetarian	2.111 (1.490–2.990)	<0.001		1.539 (1.195–1.982)	0.001	
Lacto-vegetarianism	1.956 (1.162–3.293)	0.012		1.254 (0.877–1.793)	0.215	
Lacto-ovo-vegetarianism	1.656 (1.229–2.232)	0.001		1.337 (1.051–1.701)	0.018	
Other diet	1.983 (0.796–4.940)	0.141		2.198 (0.778–6.214)	0.137	
Mixed unrestricted	Reference			Reference		

## Data Availability

Τhe study did not report any data.
